# Strengthening health quality management for cardiovascular disease, diabetes, and obesity in the United Arab Emirates: an integrated evidence synthesis

**DOI:** 10.3389/fpubh.2026.1918436

**Published:** 2026-10-28

**Authors:** Md Hafizur Rahman, Fatma Al Hamidh, Zufishan Alam, Fadumo Noor, Nazik Nurelhuda

**Affiliations:** 1 School of Health Sciences, Hamdan Bin Mohammed Smart University, Dubai, United Arab Emirates; 2 Pharmaceutical Section, Supply and Warehouse Management, Emirates Health Services, Dubai, United Arab Emirates

**Keywords:** cardiovascular diseases, diabetes, evidence synthesis, health quality management, healthcare policy, noncommunicable diseases, obesity, United Arab Emirates

## Abstract

**Introduction:**

Non-communicable diseases (NCDs), particularly cardiovascular diseases (CVDs), diabetes, and obesity, represent a major public health challenge in the United Arab Emirates (UAE). Despite strong policy and governance frameworks to address NCDs, evidence on Health Quality Management (HQM) remains fragmented, limiting the development of actionable system-level insights. Integrating empirical evidence with interest groups' experience can provide a more comprehensive and contextual understanding of HQM practices and implementation challenges. This study aims to synthesize evidence from a scoping review and interest groups' perspectives to identify systemic gaps, challenges, and recommendations for strengthening HQM in CVD, diabetes, and obesity care in the UAE.

**Methods:**

An integrated evidence synthesis was conducted using findings from a scoping review (phase 1) and interviews with 20 interest groups representing healthcare regulators and providers, academia, and patient groups (phase 2) from a multi-phase study on HQM practices in NCD care in the UAE. Data were analyzed using thematic synthesis guided by an integrated HQM framework. A mixed deductive-inductive coding and triangulation process were applied to identify areas of convergence, complementarity, and divergence between evidence sources to generate synthesized areas including gaps, challenges and recommendations.

**Results:**

Eight interconnected synthesis areas were identified: governance and implementation; access, equity and financial protection; health workforce and care culture; digital systems and data generation; care coordination and service delivery; patient-centered care and engagement; community engagement and health literacy; and multisectoral collaboration for NCD prevention. Findings revealed strong policy commitment, regulatory frameworks, and expanding digital health capacities supporting NCD care. However, persistent gaps were identified in policy implementation, care coordination, workforce competencies, digital interoperability, outcome evaluations, financial protection, equitable access, and patient and community engagement. Siloed governance and service delivery information systems, high dependency on expatriate workforce, and inconsistent cross-sector collaboration limited the translation of structural strengths into effective and equitable service delivery.

**Conclusion:**

The UAE possesses a strong policy and structural foundation for NCD quality management; however, substantial gaps remain in translating structural readiness into coordinated, patient-centered, and outcome-oriented care. Strengthening integrated governance, digital interoperability, workforce competencies, equity-focused policies, intersectoral collaboration, and performance measurement systems are essential to achieve sustainable improvements in NCD care quality and outcomes.

## 1 Introduction

Non-communicable diseases (NCDs) account for about 75 percent of global deaths. Cardiovascular diseases, diabetes, and obesity are the major drivers of this high toll (1). A large meta-analysis conducted by Emerging Risk Factors Collaboration group demonstrated that diabetes contributes to approximately 11% of cardiovascular mortality (2). Beyond biological determinants, NCDs are associated with multiple behavioral risk factors, socio-economic conditions, urbanization and demographic transitions contributing to the increasing prevalence of NCDs (3).

The prevalence of these diseases has risen significantly in the Gulf region, including the United Arab Emirates (UAE) (4).The UAE reported diabetes prevalence being 20%, and obesity more than 35%, while CVDs are the leading cause of death (approximately 40%) in the country (5–7).

In the UAE, beyond clinical impacts, NCDs also impose a substantial economic burden, estimated at around US$10.9 billion annually (about 2.7% of GDP), reflecting both healthcare costs and productivity losses. Additionally, the probability of premature mortality from NCDs remains considerable, with about 1 in 5 individuals at risk of dying before age 70 due to these conditions (8).

To address the rising burden of NCDs, the UAE has adopted a comprehensive and multi-sectoral policy response with national strategies focusing on prevention, early detection, and integrated care, including large-scale screening programs for conditions such as diabetes, cardiovascular diseases, and cancer. Government initiatives focus on addressing lifestyle and behavioral risk factors, supporting healthy environment and strengthening primary healthcare.

These efforts are supported by UAE's active collaboration with international organizations, including the World Health Organization (WHO), to enhance NCD surveillance, policy development, and overall health system strengthening. Besides the establishment of the National Action Plan (NAP) for combatting NCDs (2017–2021) aligning its efforts with WHO's Global Action Plans for NCD Prevention and Control. Federal Law No. (13) on Public Health also addresses the policy and coordination requirements for combatting NCDs in the country (9–11).

The UAE's rapidly evolving healthcare system, characterized by a predominantly expatriate population and a hybrid federal and emirate governance model along with a large private sector resembles other global health systems experiencing accelerated demographic, epidemiological, and structural transitions. High dependency on expatriate workforce and pluralistic health system structure pose challenges in care standardization, workforce competency and skills alignment, and knowledge and data sharing, representing a critical need to strengthen Health Quality Management (HQM) practices and strategies within NCD care (12).

Existing evidence on HQM for NCDs in the UAE remains fragmented and methodologically limited, constraining the development of actionable, system-level insights. Much of the current literature is dominated by cross-sectional and descriptive studies, with relatively few interventional or longitudinal designs, limiting the ability to assess effectiveness and causal impact of HQM strategies. Furthermore, key dimensions of quality particularly equity and efficiency are underrepresented, reflecting broader gaps in health systems research in the region and globally (13).

Currently, there is a critical need to complement existing evidence with insights into operational realities and implementation challenges within healthcare systems. Research has shown that policy and guideline development alone are insufficient, as gaps between policy intent and real-world implementation often limit effectiveness, particularly in complex health systems (14).

The integration of scientific evidence with interest groups' perspectives is therefore essential for addressing the complex and multi-level challenges of NCD prevention and management. NCD care involves interconnected governance structures, service delivery processes, and health system dynamics, where existing evidence is often fragmented and insufficiently translated into practice. Combining empirical research with interest groups' experiences enables a more comprehensive and contextually grounded understanding of HQM practices across the UAE health system. Moreover, triangulation of research evidence, policy guidance, and experiential knowledge enhances the validity, credibility, and practical relevance of findings, a principle widely emphasized in health policy and systems research (15). This integrative approach will facilitate the identification of systemic gaps, implementation challenges, and opportunities for improvement guiding future priorities in strengthening HQM for NCDs within the UAE health system.

Therefore, this study aims to synthesize evidence generated from phase 1 (scoping review) and phase 2 (interest group interviews) of a multi-phase study on HQM practices in cardiovascular diseases, diabetes, and obesity care in UAE, with the objective to identify systemic gaps, challenges, and opportunities for strengthening Health Quality Management practices for cardiovascular disease, diabetes, and obesity care in the UAE (16).

## 2 Methods and materials

### 2.1 Study design and data extraction

We employed an integrative evidence synthesis design and analyzed reported data from two evidence sources generated in the preceding phases of a multi-phase study by Nurelhuda and colleagues (16): (1) findings from a scoping review by Alam and colleagues (13) and (2) findings from a qualitative study of interest-group interviews by (16) Al Hamidh et al.^1^

The scoping review (phase 1) mapped the evidence on HQM practices for CVD, diabetes and obesity in the UAE between the years 2015–2025. It analyzed 54 sources comprising 31 peer reviewed interventional studies (providing evidence on HQM implementation and reported interventions and outcomes) and 23 gray literature including policy documents, guidelines and reports on system structures. Detailed characteristics of the Phase 1 evidence base are reported in the published scoping review by Alam et al. (13). While evidence from interest group interviews (phase 2) comprised perspectives and experiences of 20 key informants from relevant interest groups including health regulators (*n* = 5), healthcare providers (*n* = 6), indirect health organizations (*n* = 3), academicians (*n* = 3) and patients (*n* = 3) across the UAE health system directly engaged with HQM of CVD, diabetes and obesity. Participants were recruited using purposive and convenience sampling informed by a preceding interest-group mapping and prioritization exercise by Noor and colleagues (*Mapping Interest Groups in Health Quality Management of Cardiovascular diseases, Diabetes, and Obesity in the United Arab Emirates*, unpublished). Given the diversity of interest groups and recruitment constraints, sample adequacy was guided by information power, prioritizing participants with relevant roles and experience in NCD management rather than thematic saturation (17). Summarized characteristics of evidence stream 1 and 2 are presented in Table 1.

**Table 1:** Characteristics of evidence streams (scoping review and interest groups interviews) included in the integrated synthesis (phase 3).

Characteristic	Evidence stream 1: scoping review (13)	Evidence stream 2: a qualitative study on interest group interviews (Al Hamidh et al., unpublished)^*^
Method/design	Scoping review guided by Arksey and O'Malley framework and PRISMA-ScR	Qualitative semi-structured interest group interviews
Evidence type	Published Phase 1 scoping review findings by Alam et al. (13)	Unpublished findings from the Phase 2 qualitative study, representing the perspectives and experiences of relevant interest groups involved in HQM for CVD, diabetes, and obesity in the UAE
Evidence period	January 2015–March 2025	October–December 2025
Sources/identification	PubMed, Scopus, ProQuest, supplemented by relevant national and international gray-literature sources	Participants identified through interest-group mapping (using WHO's stakeholder mapping guidelines) and prioritization (using Mendelow's stakeholder analysis framework)
Eligibility scope	Evidence addressing health management practices related to CVD, diabetes, or obesity in the UAE	Key informants with direct experience in CVD, diabetes, and obesity related prevention or management services, programs, or policies, including their planning, implementation, or evaluation process.
Evidence/sample included in synthesis	54 sources: 31 interventional studies and 23 gray literature documents including guidelines, recommendations, and reports about HQM practices for CVD, diabetes and obesity in the UAE	20 key informants: health regulators (*n* = 5), healthcare providers (*n* = 6), indirect health organizations (*n* = 3), academicians (*n* = 3), patient representatives (*n* = 3)
Geographical coverage	Abu Dhabi, Dubai, Sharjah Ajman, Ras Al Khaima, Multiple Emirates	Abu Dhabi, Al Ain, Dubai, Sharjah, Hatta, and Dibba Al Hisn; with greater representation from Dubai

CVD, cardiovascular disease; HQM, Health Quality Management; NCD, non-communicable disease; PRISMA-ScR, preferred reporting items for systematic reviews and meta-analyses extension for scoping reviews; WHO, World Health Organization.

^*^Al Hamidh F, Noor F, Rahman MH, Alam Z, Al Bedwawi F, Shea C, Nurelhuda N. Perspectives of Interest Groups on Health Quality Management Practices for Cardiovascular diseases, Diabetes, and Obesity in the United Arab Emirates: A Qualitative Exploration. Unpublished.

No additional literature search, study screening, or formal quality appraisal was undertaken for the present Phase 3 synthesis. Screenings and evidence evaluation were performed during preceding phases (1 and 2).

Reporting of the evidence synthesis was informed by the enhancing transparency in reporting the synthesis of qualitative research (ENTREQ) checklist, where applicable, to enhance transparency (Supplementary Document 1).

### 2.2 Analytical approach for evidence synthesis

#### 2.2.1 Conceptual framework

An integrated Health Quality Management (HQM) approach was adopted by combining Donabedian's structure–process–outcome framework, the Institute of Medicine's (IOMs) six dimensions of healthcare quality (Effectiveness, Efficiency, Equity, Patient-centeredness, Safety, and Timeliness), and the WHO's health system building blocks framework as expanded by Sacks (Governance and leadership, Financing, Health workforce, Medical products, Health information systems, Service delivery, and Societal partnerships). Conceptual overlaps were accommodated within this integrated framework by allowing findings to align with more than one relevant dimension or component where appropriate and resolved further during cross cutting thematic synthesis process. Each framework contributed to a distinct but complementary level of analysis, together, these complementary frameworks provide a comprehensive, multidimensional, and system-level perspective on NCD-related HQM, ensuring that the synthesis captures both local contextual realities and internationally recognized quality standards. This enabled a structured evaluation and continuous improvement of healthcare quality across system, service delivery, and outcome domains (16, 18).

#### 2.2.2 Coding and thematic synthesis

Thematic Synthesis method was applied to integrate and compare secondary data (reported findings) from scoping review (evidence stream 1) and interest group interviews (evidence stream 2) (19).

A mixed deductive-inductive approach was applied for the coding and integration of both evidence streams. The deductive coding was guided by the integrated HQM framework, and the information was coded across the components of Donabedian, IOM and WHO's health system building blocks framework expanded by Sacks which provided an initial structure for examining the evidence. While inductive coding allowed additional patterns to emerge beyond the predefined domains. Importantly, findings that did not align clearly with the predefined Donabedian, IOM or Health System Building Blocks domains were retained during coding and considered during theme development rather than being excluded or forced into an existing category. This enabled cross-cutting and emergent areas to be incorporated into the final synthesis.

The analysis was guided by Braun and Clarke's framework (20), following six key steps:

- **Familiarization with data** through iterative review of the findings from scoping review and interest group interviews.
- **Coding:** The results sections of the scoping review and interviews (originally coded through NVIVO™) were manually and separately coded through a mixed deductive-inductive coding approach. The codes from both evidence streams were then organized within a self-developed structured evidence integration matrix for data organization across the HQM framework domains (Supplementary Document 2).
- **Initial theme generation:** Sub-themes were first generated separately for each evidence stream under each HQM domain. Data triangulation was then undertaken to compare these sub-themes across the two evidence streams and integrate them by examining areas of convergence (where findings from both evidence streams were consistent and in the same direction), complementarity (where the streams provided different but complementing insights) and divergence (where both streams reflected contrasting perspectives). Classification was based on the researchers' subjective interpretation of the findings according to these predefined conceptual definitions. Through this process initial integrated themes were generated for each HQM domain (Figure 1) (15).
- **Theme review and development of cross-cutting thematic areas:** following the triangulation, the initial integrated themes were reviewed across the predefined HQM domains for identification of recurrent patterns, relationships and issues. Findings that did not fit predefined domains were retained and considered in this step, to allow emergent thematic areas to be incorporated into the synthesis. Conceptually related findings were clustered into broader cross-cutting themes reflecting thematic areas rather than being restricted to pre-defined domains.
- **Theme definition:** The broader themes under each thematic area were refined and defined to clearly reflect the integrated content and its relevance to HQM of CVD, diabetes and obesity.
- **Report production:** The final thematic areas and synthesized themes were presented through description and interpretation of the integrated evidence from both evidence streams. As illustrated in Figure 1, the resulting thematic areas formed the basis for identification of recurring gaps, challenges and synthesized recommendations for HQM of CVD, diabetes and obesity in the UAE (20). The recommendations were formulated to directly address the identified gaps and challenges based on researchers' reflexivity and understanding of the subject matter.

**Figure 1:**
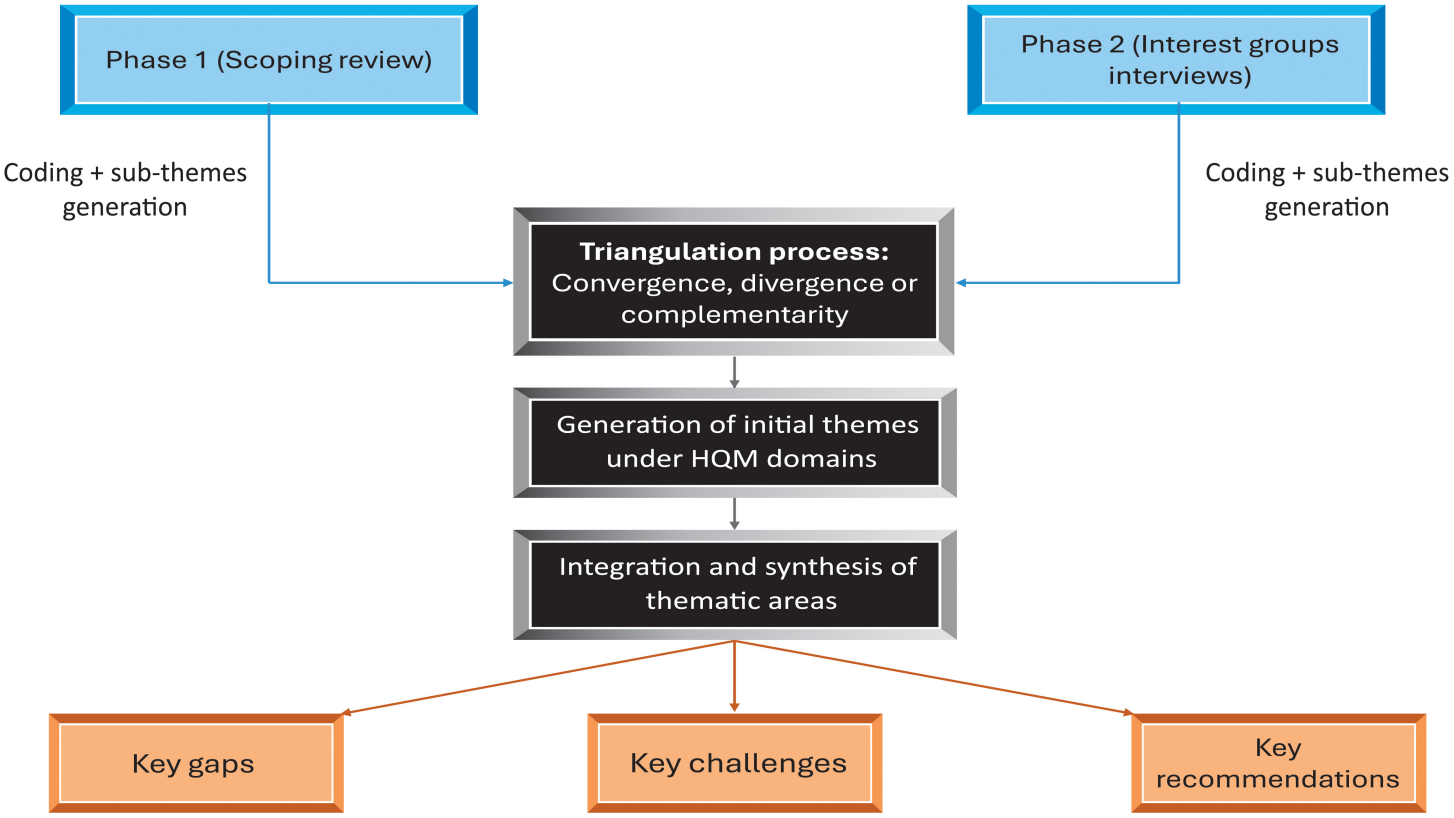
Steps of the thematic synthesis from integrated evidence (phase 1 and phase 2).

This comparative approach between the two evidence streams improved rigor highlighting implementation gaps between policy and practice, differences between academic research priorities and actual system needs, and overlooked challenges in implementation.

To ensure rigor, two research team members (FA and MHR) collaboratively performed coding and synthesis. Researcher reflexivity was considered throughout the synthesis. Both the coders had adequate training and experience in qualitative research methods, complemented by subject-matter expertise in non-communicable disease research, programs, policies and UAE health systems supported by their professional and academic background. Disagreements were resolved through discussion and iterative refinement of codes and themes until consensus was achieved on final themes. A reflexive journal was maintained throughout the synthesis process to document analytical decisions and reflections.

## 3 Results

The findings are organized into eight synthesized thematic areas across health system levels. These are presented according to: (1) synthesized theme; (2) the relationship between evidence streams through convergence, complementarity, or divergence, (3) alignment with the Institute of Medicine quality dimensions and the Donabedian Model, and (4) identified gaps, challenges, and improvement recommendations.

Summary findings from phase 1 and phase 2 evidence streams are included in Supplementary Document 3.

### 3.1 Governance and implementation

- *Strong policy driven governance with inconsistencies in implementation, outcome evaluation, collaboration, and system capacities limiting their effectiveness*.

Within this synthesis area, the integration revealed a convergence relationship; both Phase 1 and 2 showed relatively strong policy and strategic direction with strong governance foundation. Complementarity was also evident as Phase 1 emphasized strong policy and regulatory frameworks, while Phase 2 further revealed implementation, coordination and service capacity gaps that limited effective translation into practice. A minor divergence also emerged regarding the evaluation practices. Phase 1 described lack of outcome evaluation particularly WHO quality-of-care indicators such as patient-reported outcomes, access and resource-use measures. On the other hand, Phase 2 showed that despite lack of policy impact evaluations, performance monitoring for NCD related policies and practices using relevant KPIs is in place.

Key gaps included policy–practice misalignment and limited outcome evaluation using standardized metrics. While identified challenges were related to policy and research translation, fragmented governance and coordination across sectors and levels of care, and limitations in workforce and system integration capacities. To address these gaps and challenges, it is important to strengthen governance-to-implementation pathways through operational frameworks, accountability mechanisms, and policy impact evaluation, alongside enhancing cross-sector collaboration to support coordinated policy and knowledge translation.

This synthesis area primarily aligns with the governance and leadership component of health system building block and the IOM's quality dimensions of effectiveness, safety, and timeliness. It reflects all domains of the Donabedian Model including structure, process, and outcome measures.

### 3.2 Access, equity, and financial protection

- *Apparent adequacy in population-level coverage masks inequities in access and financial protection associated with variations in insurance design and geographical disparities in service reach*.

The synthesis of this theme revealed complementarity with minor divergence across both evidence streams where national level evidence indicates financial protection at population level as indicated through national reports (Phase 1), but Phase 2 findings uncover inequitable financial insurance coverage limiting access and equity along with geographical disparities in resource management particularly access to digital tools.

Key gaps and challenges included fragmented insurance systems across population groups and emirates, inequitable access to medicines, diabetes self-management tools, and digital infrastructure, as well as limited equity-focused research. Combined population-level reporting was also found to mask inequities in healthcare access and resource distribution.

Addressing this requires re-designing insurance policies to include unified and standardized insurance plans across the UAE and improving access to low-income expatriates like laborers. Importantly insurance policies and resource planning should be equity driven while research on financial barriers and access to CVD, diabetes and obesity care among low-income expatriates needs to be expanded for designing evidence-based financing models.

These findings relate to financing component of the health system building blocks and to equity, effectiveness, efficiency and timeliness dimensions of quality of care and structural measures of Donabedian model.

### 3.3 Health workforce and care culture

- *Established staffing structures with variability in workforce capacities, competencies, and evidence, affecting responsiveness of care*.

Finding from both evidence streams in relation to this synthesis area showed complementarity relationship. Phase 1 identified that workforce policies and staffing requirements exist while Phase 2 interviews revealed gaps in in workforce capacities particularly workload and time management constraints, limited communication, competencies and skills that limit providers from engaging patients and delivering patient-centered care.

Beyond workforce structure and requirements, workforce quality emerged as a critical issue.

Thereafter the key gaps include workforce shortages in specialized NCD management and public health alongside limitations in workforce skills specifically in relation to their patient-centered communication and engagement skills.

These gaps are can be associated to insufficient evidence and research focus on workforce challenges in NCD care and challenges in translating the policy and workforce strategies into focused training and capacity building initiatives.

Importantly, strengthening workforce competencies requires structured capacity building training programs for NCD prevention and management along with enhancing research capacities to identify and address workforce challenges in different settings to facilitate evidence-based workforce planning.

This synthesis area strongly relates to health workforce building block of the health system and affects patient-centeredness, effectiveness, efficiency and timeliness of care delivery, while representing structural and process components of Donabedian model.

### 3.4 Digital systems and data generation

- *Advancing digital systems and data generation with gaps in research scope, interoperability, and equity affecting care coordination and system wide effectiveness*.

Digital and data infrastructure emerged as a key factor in both enabling and limiting NCD quality management. While digitalization and data infrastructure was identified as an enabler, growing and advancing in both scoping review and interest groups interviews showing convergence relationship, findings from the interviews showed complementarity uncovering gaps in digital system and data integration across the healthcare providing organizations and sectors.

Findings revealed fragmented system integrations which affect smooth and safe patient information transitions across settings. Furthermore, gaps include limited data generation specifically in obesity research and outcome level data such as patient related outcomes and reported experience measures. Additionally, inequities in accessing advanced digital tools (e.g., for diabetes self-management) were also identified across settings particularly in remote areas.

These gaps mainly related to isolated digital systems and limited interoperability, prioritizing CVD and diabetes research over obesity, and inequitable distribution and implementation of digital advancements across emirates due to budget limitations.

Strengthening national level system integration and digital interoperability for data integration is required across healthcare providers for enhanced coordination and care continuity. Expansion of data coverage for underrepresented conditions (e.g., obesity) and strengthen data generation on outcome measures alongside equitable deployment of digital advancements within and across emirates are key improvement needs in this area.

From the WHO health system building blocks perspective, these findings largely reflected the health information systems (HIS) component emphasizing the importance of integrated, accessible, and equitable data systems for informed decision-making. These findings were particularly relevant to the IOM quality dimensions of effectiveness, efficiency, safety, equity, timeliness, and patient-centeredness. From a Donabedian perspective, the findings mainly reflected structural measures.

### 3.5 Care coordination and service delivery

- *Inconsistencies in patient follow-ups, referrals, and care coordination across settings influence continuity of care, while overreliance on disease-focused care limits preventive approaches*.

Triangulation and synthesis of findings under service delivery domain indicated complementarity with minor divergence relationship between the scoping review findings which showed greater emphasis on and prioritization of care coordination through policy focus, while interview findings complementing and slightly diverging by revealing implementation gaps in care integration and continuity. Findings also converged in terms of shared emphasis on disease management over preventive care.

The identified gaps in relation to service delivery included inconsistent care integration; coordination and continuity between public and private providers including patient follow breakdowns and gaps in mitigation strategies to accommodate missed patients' appointments. Disparities in care coordination across geographical areas were also identified as Dubai -based healthcare providers were found to be more organized and coordinated in NCD service delivery compared to those in remote areas. In addition to limited prevention emphasis at point of care.

These gaps can be associated to weak coordination mechanisms within and across care settings and levels due to siloed care delivery systems in the country and limited provider capacity and skills for preventive care. Suggesting a need for strengthened patients follow up mechanisms through flexible, adaptive, and optimized appointment systems alongside strengthened system integration across providers for coordinated care. Importantly, provider capacity building for integrating prevention models into care delivery should also be developed.

This synthesis area represents the service delivery component of WHO's health system building blocks and Donabedian's process measures. The findings relate to IOM's quality of care domains of effectiveness, efficiency, timeliness.

### 3.6 Patient centered care and engagement

- *Emerging patient centered care models with gaps in policy integration, evaluations, digital usability and patient engagement in care*.

Findings from both evidence streams demonstrated convergence with complementarity relationship.

Both evidence streams emphasize the growing focus on patient empowerment, personalized care and shift toward patient-centered approaches in NCD care delivery. Phase 2 further reflects persistent gaps in practice including communication, engagement, and responsiveness in care.

Key identified gaps were digital usability and literacy challenges by the patients for the use of self-management tools and digital platforms, gaps in physician-patient communication, lack of emotional support and responsiveness by the physicians, and lack of patient engagement in care and decision-making.

These gaps could be particularly emerging from limited consultation time with patients, workforce pressures, and gaps in provider competencies related to patient centered care. Additional challenges included limited integration of patient education and psychological support into care pathways, inadequate structural and policy support for patient-centered care, insufficient inclusion of outcome metrics and patient reported care experience measures in policy documents and research. Furthermore, limited digital capacity building for specific patient groups e.g., geriatrics further constrained engagement with digital health services. Eventually these challenges undermine patient centered care.

In line with these gaps, stronger policy and administrative support to optimize consultation time and care processes that support adequate attention to patients are required. Additionally, embedding patient-centeredness indicators into policy and evaluation frameworks, and enhancing provider communication and patient engagement skills through structured training and capacity building initiatives are needed.

From the WHO health system building blocks perspective, the findings reflected the service delivery component, emphasizing the importance of responsive, patient-centered care delivery. From a Donabedian perspective, they relate to both process and outcome measures, while they are particularly relevant to the IOM quality dimensions of patient-centeredness, safety, and effectiveness.

### 3.7 Community engagement and health literacy

- *Emerging community engagement is characterized by gaps in health literacy, fragmented and inequitable outreach across low-income worker populations*.

Both evidence streams showed convergence with complementarity regarding community engagement and health literacy. Both phases revealed shared emphasis on equity gaps affecting low-income expatriates. Phase 1 findings revealed underrepresentation of diverse and low-income populations in research while Phase 2 interviews further revealed limitations in engaging them in health awareness programs highlighting fragmented outreach to these population groups and inadequate adaptation of educational and health literacy programs for low-income laborers.

Overall, the synthesis indicated that participatory models are emerging through community advocacy within policy and service design, however, these remain constrained by gaps in underrepresentation of low-income expatriates in research and inadequate community awareness, engagement, and inadequate culinary skills for healthy food preparation. These gaps were linked to limited and inequitable outreach resources particularly for segmented communication and awareness programs, and cultural and language barriers affecting engagement of low-income workers.

The findings highlight the need to strengthen community participation and representation in research and expand community awareness and health literacy programs through adequate resource allocation and culturally adaptive approaches targeting underserved populations.

The findings mainly reflected the service delivery component of WHO's health system building block, while also aligning with the expanded Sacks framework emphasizing community engagement, societal partnerships, and community participation in health systems strengthening. The findings relate to Donabedian's process measures, and IOM's quality dimensions of patient centeredness (community-responsive approaches), equity (disparities in outreach and representation), and effectiveness (healthier behaviors, prevention practices, and informed self-management).

### 3.8 Multisectoral collaboration for prevention of NCDs

- *Multi-sectoral prevention exists in policy with inconsistent coordination limiting the system level prevention practices*.

The integrated findings demonstrated that multi-sectoral collaboration for the prevention of NCDs is strongly emphasized at policy level in the UAE. This was revealed by Phase 1 findings that showed policy directions advocating for cross-sector collaboration for prevention activities particularly the UAE NCD National Action Plan (2017–2021). However, these efforts were found to be isolated rather than coordinated. Phase 2 findings further expanded this understanding by revealing the evolving collaboration across sectors such as between healthcare providers, regulators, academia, education sector and local municipalities. Despite these efforts, interviews also highlighted fragmented and short-term intersectoral collaborations. Together, these findings show a minor convergence and complementarity relationship between both evidence streams.

Key gaps include fragmentation in intersectoral collaboration and isolated prevention initiatives that are event or program based, while the key challenges include limited coordination across policy, planning and service delivery, project-based collaboration with lack of long-term engagement, and limited data sharing across sectors.

Addressing these challenges requires strengthening cross-sector coordination through structured engagement, collaboration focal points, integrated governance platforms, and data sharing systems.

Cross-sectoral collaboration for NCD prevention was linked to multiple IOM quality dimensions including effectiveness and safety (through health promotion and preventive practices), efficiency (through cross-sector coordination and integrated platforms), and patient centeredness and equity (through community and patient and patient engagement). The findings relate to Donabedian's structure and process measures and align with the “Societal partnerships” health system building block expanded by Sacks.

Table 2 shows the generated sub-themes across both evidence streams (Phase 1 and Phase 2) and integrated (synthesized) themes under the identified thematic areas.

**Table 2:** Synthesized themes across thematic areas generated from key findings of Phase 1 (scoping review) and Phase 2 (Interest group interviews).

Thematic area	Phase 1 (scoping review) sub-themes (13)	Phase 2 (interest group interviews) sub-themes (Al Hamidh et al., unpublished)^*^	Synthesized (integrated) themes
Governance and Implementation	•Strong policy-driven governance and strategic prioritization of NCD care •Regulatory frameworks defining standards of care and safety requirements •Emphasis on access and timeliness within policy direction •Gaps in embedding patient-centeredness within policy frameworks •Policy documents emphasized structure and process more than outcome monitoring •Absence of UAE-based studies or policy documents applying WHO quality-of-care indicators such as patient-reported outcomes, access and resource-use measures	•Policy-practice and accountability gaps •Care standardization, quality assurance, performance oversight, accountability, and strategic planning •Evolving but fragmented system coordination and collaboration across sectors •Infrastructure, capacity, and integrated system development needs •Policy impact evaluation, and translation into routine practice are uneven. •Performance monitoring exists using relevant policy KPIs	*Strong policy driven governance with inconsistencies in implementation, outcome evaluation, collaboration, and system capacities limiting their effectiveness*
Access, equity, and financial protection	•Gaps in addressing financial protection within research evidence •National report indicates financial protection at population level •Geographic concentration but emerging expansion of research scope •Imbalance in disease and risk factor representation in research •Limited and uneven attention to equity across evidence sources	•Inequitable insurance designs and financial protection limit access and equity •Geographical disparities in resource management and access including digital tools	*Apparent adequacy in population-level coverage masks inequities in access and financial protection associated with variations in insurance design and geographical disparities in service reach*
Health workforce and care culture	•Availability of policies addressing human resource and staffing requirements •Lack of research focus on structural dimensions including workforce capacity, staffing distribution, skill mix, or organizational readiness	•Workforce capacity and competency gaps affecting NCD care quality •Gaps in patient-centered communication and engagement affecting quality of care and patient experience	*Established staffing structures with variability in workforce capacities, competencies, and evidence, affecting responsiveness of care*
Digital systems and data generation	•Limited data coverage across conditions e.g., obesity •Evolving data and expanding research scope post-COVID-19 •Growing focus on digitalization in health research and care delivery •Overreliance on process level data with limited patient reported experience and outcome data •Evidence is still limited on implementation scale, user adherence, patient satisfaction, interoperability, and long-term sustainability	•Advancing digital enablement (dashboard, remote monitoring, AI predictive models etc.) for NCD prevention and care with equity challenges •Fragmented system and care Integration requiring strengthening	*Advancing digital systems and data generation with gaps in research scope, interoperability, and equity affecting care coordination and system wide effectiveness*
Care coordination and service delivery	•Partial integration of multiple disease management approaches in research •Policy focuses on coordinated care delivery •Predominant focus on disease management over prevention •Care processes across the NCD pathway, spanning screening, diagnosis, management, emergency care, and digital monitoring. •Inconsistent evidence on care coordination, referral pathways, and continuity of care.	•Disparities in care coordination across settings •Digital integration challenges across service delivery settings •Inconsistencies in patient follow ups and challenges in referral systems across settings (public vs. private)	*Inconsistencies in patient follow-ups, referrals, and care coordination across settings influence continuity of care, while overreliance on disease-focused care limits preventive approaches*
Patient centered care and engagement	•Patient-centeredness reflected through clinical and behavioral measures •Evidence gap in patient-reported outcomes or patient experience measures •Evolving emphasis on personalized and patient empowerment-focused care approaches •Gaps in structural and policy support for patient-centeredness •Underrepresentation of patient-centeredness in literature	•Emerging patient-centered care models •Gaps in patient engagement, communication, emotional support, and responsiveness •Patient-centered practices enhance safety of care through education •Digital usability challenges by patients	*Emerging patient centered care models with gaps in policy integration, evaluations, digital usability and patient engagement in care*
Community engagement and health literacy	•Underrepresentation of diverse populations in research •Literature focus on lifestyle and behavioral interventions within effectiveness domain •Gaps in equity, adaptation, and reach across population groups particularly marginalized groups	•Emerging participatory models through community advocacy within policy and service design •Inadequate health awareness programs for low income •Emerging culturally sensitive nutritional guidelines •Inadequate community health literacy and skills particularly culinary knowledge and skills •Fragmented and under-resourced (limited budget and manpower) community outreach particularly for low-income groups	*Emerging community engagement is characterized by gaps in health literacy, fragmented and inequitable outreach across low-income worker populations*
Multisectoral collaboration for prevention of NCDs	•Policy emphasis on multi-sectoral collaboration for prevention •Prevention interventions described as isolated initiatives rather than coordinated, multisectoral prevention strategies across sectors	•Evolving cross-sectoral and multi-level collaborations •Prevention programs are multisectoral, involving actors across health system, education, municipalities, urban planning, and community stakeholders •Fragmentation in intersectoral collaboration mechanisms	*Multi-sectoral prevention exists in policy with inconsistent coordination limiting the system level prevention practices*

^*^Al Hamidh F, Noor F., Rahman MH, Alam Z, Al Bedwawi F, Shea C, Nurelhuda N. Perspectives of Interest Groups on Health Quality Management Practices for Cardiovascular diseases, Diabetes, and Obesity in the United Arab Emirates: A Qualitative Exploration. Unpublished.

Summary of key findings across the synthesized areas are presented in Figure 2. Detailed evidence synthesis matrix is available as Supplementary Document 2.

**Figure 2:**
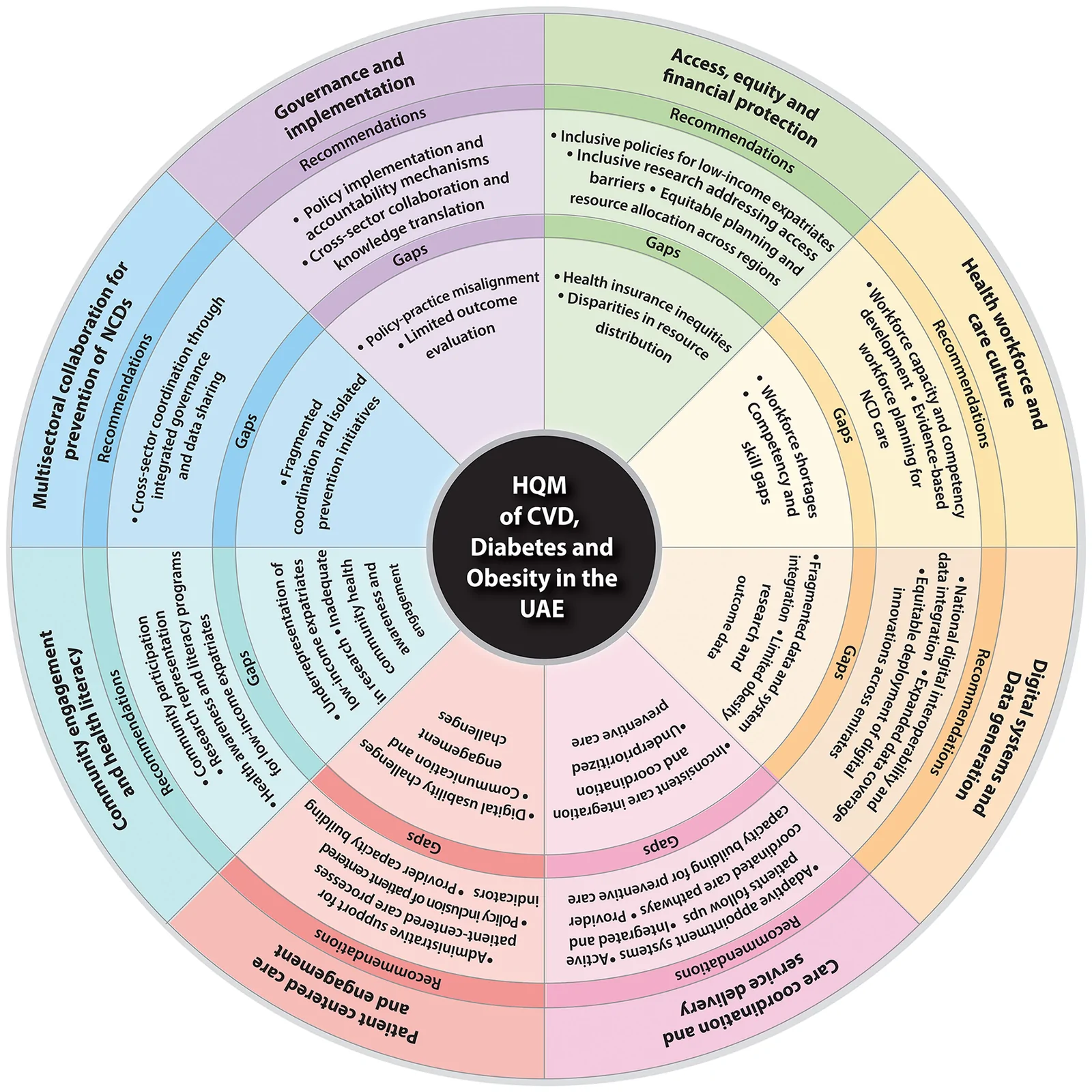
Summary of key synthesized findings (gaps and recommendations) from integrated evidence, according to thematic areas.

## 4 Discussion

### 4.1 Discussion of key findings

This study provides a system level analysis of HQM practices in CVD, diabetes and obesity prevention and care in the UAE integrating evidence from scientific literature and the experiences of the relevant interest group members.

Key insights from the synthesis indicate strong governance through policy commitment, regulatory frameworks, quality infrastructure and expanding digital health capacities to support NCD prevention and care. However, gaps persist in translating these policy strengths and program capacities into consistently coordinated, equitable, patient centered and outcome-oriented care delivery.

These findings highlight the imbalance between structural readiness and operational efficiencies consistent with Donabedian's model suggesting that structural strengths alone may not translate into effective processes and outcomes when systemic and operational gaps persist (21). Policy intentions often fail due to inadequate implementation mechanisms, weak leadership engagement, and insufficient system-level support (14). While our findings show a strong leadership commitment, there are deficiencies in other components of the NCD management system in the UAE particularly in workforce capacities, resource planning, financial protection, coordination mechanisms, digital interoperability, and community and patient engagement.

Workforce competencies emerged as a critical factor for delivering responsive and patient-centered NCD care. Although workforce requirements are established, specific competency frameworks for NCD care and patient engagement remain limited. Like Gulf Cooperation Council (GCC) countries, the UAE health system is highly dependent on expatriate workforce, creating challenges in standardizing practices, maintaining expertise and ensuring continuity of care amid high staff turnover rate post COVID-19 (12). These workforce constraints are further challenged by growing healthcare demands and limited consultation times. A report from Mohammed Bin Rashed School of Government revealed care responsiveness concerns in the UAE, it showed that more than 60% of patients surveyed indicated doctors do not spend adequate time with them during visits (22). Consequently, proactive preventive care services such as lifestyle counseling and patient-centered primary care become limited, making the current system predominantly reactive and disease centric.

Furthermore, as discussed by Armocida and colleagues that structural strengths should leverage the shift toward more coordinated and data driven care delivery to improve care continuity. This addresses the care coordination gaps as revealed by our study findings, through more proactive patient follow-up, flexible and adaptive appointment systems that are supported by digital technologies like artificial intelligence (AI) enabled scheduling systems and remote consultations where applicable (23).

With regards to data generation, monitoring and evaluation, the current evidence-base is predominantly clinical and process oriented, with limited emphasis on outcome and HQM evaluations, financial disparities, workforce challenges, economic evaluations, and access to essential medical products across different settings. Current monitoring systems in the UAE are variable with inconsistent data collection methods and reporting standards. These findings are supported by a multi-country study conducted in Europe that highlights the importance of establishing robust monitoring and NCD care surveillance systems to guide evidence-based policymaking and improve health system responsiveness (23).

Existing literature further highlights the lack of standardized tools and robust metrics to evaluate the implementation and impact of quality management systems, resulting in gaps in continuous quality improvement processes (14). These findings align with our results demonstrating variability in monitoring practices and fragmented data systems. Emerging evidence further emphasizes the importance of standardized and harmonized quality indicators aligned with the values and needs of different interest groups in NCD management. Besides investment in system wide measurement and feedback systems for NCD care supported by integrated digital health infrastructures and stronger governance mechanisms to enable coordinated monitoring and data sharing. Importantly, monitoring frameworks should extend beyond traditional clinical indicators to incorporate broader quality and equity dimensions, including patient-reported outcomes, access disparities, and social determinants of health, to support more comprehensive and patient-centered evaluation of NCD care (23).

Our findings are also consistent with evidence from organization of economic cooperation and development (OECD) countries, where health systems demonstrate strong structural capacities with implementation challenges. Increasing healthcare demand, financial pressure and workforce shortages continue to challenge sustainable and efficient care in these countries, especially in the post COVID-19 era (24). Similar financial protection and access challenges were reported by GCC countries despite expanding insurance schemes, due to heterogenous implementation of health reforms, diversity in service delivery and emerging healthcare challenges (25).

In the UAE, fragmented insurance designs and variable benefit packages, mainly due to domination of private sector, appear to contribute to inequities in healthcare access, particularly for preventive services (25). These findings are further supported by a UAE report highlighting healthcare affordability, disparities, and mixed perceptions regarding access across socio-economic groups (22).

Given the impact of healthcare access and financial inequities on the effectiveness of care and treatment outcomes, the WHO advocates for strengthening universal health coverage (UHC) especially in primary care to reduce the out-of-pocket payment and NCD burden particularly among vulnerable populations (10). Outcome-based and value-based financing models may offer promising approaches for improving efficiency and value for money. However successful implementation requires adaptation to the local context and organizational readiness in terms of leadership commitment, regulatory frameworks and strong data systems for monitoring and verification purpose (26). While these enablers exist in the UAE's NCD system, digital interoperability and data integration challenges as identified in our findings may constrain effective implementation of these models.

Like many other developed and GCC countries, the UAE, despite having advanced digital infrastructure, continues to face challenges in fragmentation of electronic medical records (EMR) systems and data silos mainly across public and private sectors resulting in unreliable use of health information (27). Our findings further highlight implementation and user-level barriers including limited engagement, usability and digital literacy among both provider and the public, emphasizing the need for greater involvement of interest groups across implementation stages. Tailored community workshops and awareness programs for older adults and their caregivers delivered through primary healthcare centers and community-based channels such as social organizations, community centers, and home-based services, can support enhancing the digital literacy among these groups (28).

Strengthening interoperability between electronic health records, data registries, and public health databases is critical for improving real-time monitoring and informed decision-making at national level (23).

Importantly, data fragmentation and siloed systems are extended challenges across sectors involved in coordinating NCD prevention efforts. Bridging these gaps requires stronger “Health in All Policies” (HiAP) and a “whole of government and society” strategies to integrate NCD goals across the sectors (10).

Furthermore, our findings support the evidence from GCC countries that insufficient collaboration between academic institutions, healthcare policy makers and practitioners is hampering the ability to use evidence-based findings to inform policies and resource allocation (27).

While cross-sectoral collaboration is evolving with shared focus on population level prevention strategies, these efforts remain inequitable and inadequate in outreach and adaptations for low-income expatriates such as laborers. Targeted NCD-related health promotion and awareness interventions offered through multilingual and visually supported communication, alongside strategic partnerships with labor camps and employers across the country, can help improve outreach, health literacy, and engagement in preventive health practices among these vulnerable groups (29).

Overall, these findings position the UAE within a broader global NCDs management system while also providing opportunities to leverage its relatively strong structural foundation to improve the HQM of NCDs in the country.

### 4.2 Policy, practice and outcome level implications

The integrated synthesis underscores the need for strategic shift toward enhanced system integration, interoperability, and workforce capacity development for NCDs in the UAE through integrated governance models.

A key priority is the development of interoperable health information systems that enable seamless data exchange across providers, payers, and public health entities, supported by standardized national data systems and robust governance policies. Strengthened accountability mechanisms and integrated governance platforms are essential to facilitate coordination, data sharing and evidence informed decision making.

Strengthening cross-sector collaboration through structured engagement mechanisms and stronger partnerships between policymakers, healthcare organizations, academia, and community sectors is also critical to improve policy implementation and knowledge translation. Strengthened public–private partnerships (PPPs) can play a pivotal role in operationalizing this strategic shift by bridging fragmentation across sectors and enhancing system integration as well as through performance-based contracting, where clear roles, shared metrics, and outcome-driven incentives promote integration across levels of care (12, 22).

Policy reforms should facilitate equity-focused planning and resource allocation to address geographical disparities in healthcare resources including access to advanced digital technologies for NCD prevention and care across and within emirates.

Structural reforms should additionally prioritize financial policy reforms that improve healthcare affordability and equitable access to preventive and chronic care services, particularly in remote areas and among low-income expatriate populations (25).

Sustained investment in health workforce capacity, particularly acquiring sufficient multidisciplinary workforce with the right skills and competencies in NCD care, prevention and digital health is equally important to address current service delivery constraints. Given the UAE's growing dependence on expatriate workforce, enhancing staff retention policies and investment in frameworks for NCD capacity building are essential to preserve and upskill the workforce capacities (24, 27).

Collectively, these structural enhancements will create the foundational conditions necessary for translating policy commitments into coordinated, high-quality care delivery.

At the practice level, strengthening HQM requires translating policy intent into consistent, integrated care delivery processes. This requires enhancing multidisciplinary coordination and communication referral pathways, and information transfer across settings. Clinical practice should move beyond disease focused management to integrate preventive care into routine practice. This is important for improving effectiveness of care and long-term outcomes.

Furthermore, healthcare providers, particularly public providers and those in remote areas, should optimize appointment systems and patient follow-up mechanisms to strengthen care continuity and responsiveness. Workforce capacity building should prioritize competencies in patient-centered communication, patient engagement, and preventive care counseling. Additionally, stronger administrative and operational support is needed to reduce workload pressures for healthcare providers and allow adequate consultation time for meaningful physician–patient interactions and responsive care delivery.

Improving digital health literacy and usability, particularly among older and vulnerable patient groups, is also critical to enhance engagement with self-management technologies and digital health services. In parallel, strengthening community outreach and cross-sector collaboration through structured, coordinated and tailored health promotion initiatives is essential particularly for underserved populations such as low-income laborers.

Health system efforts should also be directed toward building research capacities to carry out effective health system research, alongside enhancing system integration for optimal data utilization for NCDs research.

Finally, embedding continuous quality improvement (CQI) mechanisms such as feedback loops, and performance benchmarking will be essential to reduce variability in implementation and ensure the sustained translation of policies into high-quality practice.

At the outcome level, strengthening HQM requires advancing comprehensive and standardized measurement systems to evaluate the effectiveness of the NCDs service delivery including patient-reported outcomes and equity measures beyond the evaluation of clinical indicators.

Improving system efficiency and sustainability through better chronic disease control, reduced avoidable admissions, optimized resource and service utilization, and robust evaluations of healthcare quality indicators are essential. The integration of interoperable data platforms and real-time dashboards can further support continuous monitoring, high-quality data generation, and evidence-based decision-making. Importantly this should be supported by the institutionalization of national performance monitoring frameworks incorporating WHO-aligned quality indicators along with audit systems to enable effective and consistent performance evaluations for sustained improvement in NCD care (14, 23).

### 4.3 Future research directions

Our findings highlight several future research priorities. First, equity focused NCD research is critically needed to evaluate disparities in care access for CVD, diabetes and obesity across low-income expatriate workers, geographical regions and socio-economic groups. Importantly, future research should prioritize needs assessments exploration of care seeking behaviors among these vulnerable populations to understand educational and health literacy requirements to inform effective interventions.

Implementation science research and policy impact assessments are required to evaluate how current NCD policies, frameworks and interventions are being translated into practice.

Given the scarcity of data on cost effectiveness of NCD interventions, economic evaluations in this area are required to strengthen efficiency dimension of NCD care quality which is already under-represented in current evidence base.

Longitudinal and interventional studies are also required to evaluate the effectiveness of multidisciplinary care pathways, prevention strategies, and community-based interventions in improving NCD outcomes. Similarly, attention is required to health workforce research examining skill mix, communication competencies, workload pressures, and retention dynamics, particularly in the context of the UAE's reliance on the expatriate workforce.

Finally, future studies should investigate the effectiveness of intersectoral partnerships, including collaborations in supporting integrated and preventive approaches for NCD management.

### 4.4 Strengths of the study

This study has several strengths; it adopts a multi-phase integrative evidence synthesis approach combining findings from a scoping review with interest groups' perspectives enabling a more comprehensive understanding of HQM of complex issues like NCDs and addressing the limitations of fragmented evidence in the field.

The use of triangulation across multiple evidence streams enhances methodological rigor and trustworthiness of findings particularly credibility, confirmability and dependability supported by transparent and structured synthesis approach.

Besides, engaging multiple interest groups perspectives with evidence base provides a depth of insights and contextual realities not captured in literature.

The application and integration of international healthcare quality frameworks like Donabedian, IOM's six quality domain and health systems building blocks expanded by sacks provides a robust conceptual foundation for analysis ensuring comprehensive multi-level system evaluation of HQM practices and supporting actionable policy relevant insights.

Additionally, this study addresses the gaps in the current NCD evidence base in the UAE by addressing underexplored quality domains particularly equity and efficiency.

### 4.5 Limitations

Despite the strengths discussed, there are some limitations that should be acknowledged.

The evidentiary strength of the integrated findings should be interpreted in light of limitations within both evidence streams. First, the synthesis is partly constrained by the existing heterogenous evidence base of the scoping review (Phase 1), which is predominantly composed of cross-sectional and descriptive studies with limited interventional and longitudinal research, restricting the ability to draw causal inferences on HQM effectiveness. Hence, Phase 1 evidence stream may be subject to publication and document availability bias, potentially limiting the evidence captured.

Second, while interest groups perspectives provided valuable contextual insights, Phase 2 had limitations related to key informant recruitment. Although participants represented a range of institutional and interest groups backgrounds, representation was not comprehensive across all emirates or interest group categories, with some groups, such as community and financial organizations not being represented. As a result, the findings may not fully capture the diversity of experiences across all healthcare settings and population groups in the UAE.

The purposive and convenience sampling of participants and organizations may have introduced some selection and institutional biases. These were addressed through diverse institutional representation, confidentiality and anonymity. Researcher interpretive bias is also possible given the qualitative nature of the synthesis. These factors may partly affect the results and conclusions; however, this was mitigated through collaborative coding, consensus-based resolution of disagreements, and reflexive documentation throughout the synthesis process.

Additionally, the integration and triangulation approach enhances the depth and credibility of the findings.

## 5 Conclusion

This study advances the understanding of HQM for CVD, diabetes and obesity care in the UAE through an integrated analysis of empirical evidence and interest groups perspectives.

Findings highlight that while the UAE has strong NCD governing policy and structural foundation in place, multiple gaps across care delivery and capacities, integration, coordination, and patient and community engagement continue to limit the translation of this structural readiness into high-quality management. The study underscores the need for a strategic shift toward integrated, patient-centered, and collaborative equity-focused models of care, supported by interoperable health systems, strengthened workforce competencies, and comprehensive benchmarked NCD performance measurement systems. By identifying critical gaps across structure, process, and outcome domains, this research provides actionable and context-sensitive insights to inform policy and practice of NCD management in the UAE. Ultimately, aligning structural capacity with effective implementation through more accountable and coordinated actions is critical to achieve sustainable improvements in NCD care and supporting the UAE's goal of positioning itself as a high-performing health system.

## Data Availability

The original contributions presented in the study are included in the article/Supplementary material, further inquiries can be directed to the corresponding author.

## Footnotes

1 Al Hamidh and *colleagues (Perspectives of Interest Groups on Health Quality Management Practices for Cardiovascular diseases, Diabetes, and Obesity in the United Arab Emirates: A Qualitative Exploration. Unpublished)*.
