# Supplementary material for: Strengthening health quality management for cardiovascular disease, diabetes, and obesity in the United Arab Emirates: an integrated evidence synthesis
Source: Front Public Health. 2026 Oct 28;14:1918436. doi: 10.3389/fpubh.2026.1918436 (PMC13752134; doi:10.3389/fpubh.2026.1918436)
Supplement: Supplementary file 1 [file Supplementary_File_1.docx]

Supplementary Material

# Overview of findings from Phase 1 and Phase 2 evidence streams

**Findings from Phase 1 (Scoping Review)- by Alam et al (2026)**

*Alam Z, Sinha K, AlBedwawi F, Osman H, Rahman MH, Noor F and Nurelhuda N (2026) Health quality management practises for cardiovascular diseases, diabetes, and obesity care in the UAE: a scoping review. Front. Public Health 13:1703376. doi: 10.3389/fpubh.2025.1703376*

The scoping review identified a policy-driven and structurally grounded approach to NCD care, with strong emphasis on governance, regulation, and strategic prioritization. Evidence indicates that national policies and guidelines provide a robust foundation for NCD management, particularly in defining standards of care, safety requirements, and access to services. However, important gaps remain in the translation of policy into practice, especially in embedding patient-centered care and standardized performance indicators within the system.

From a structural perspective, health information systems are evolving, with increasing attention to digitalization and data-driven approaches, particularly in the post-COVID-19 context. Despite this progress, significant data gaps persist, particularly in efficiency measurement, obesity-related risk factors, and comprehensive system performance evaluation. Workforce-related policies are present, but there is limited empirical research addressing workforce challenges, indicating a gap between policy intent and research focus.

At the process level, findings highlight partial integration of care delivery models, with growing attention to patient-centered and coordinated care approaches. However, there is a predominant focus on disease management rather than prevention, and limited emphasis on societal partnerships or cross-sectoral collaboration.

In terms of outcomes, the evidence base is characterized by a dominance of effectiveness-focused research, with limited interventional and long-term studies. Efficiency is underexplored, with inconsistent measurement and limited cost-effectiveness analyses. Equity remains unevenly addressed, with geographic concentration of research and underrepresentation of diverse populations, particularly expatriate groups. While patient-centeredness is reflected in clinical and behavioral interventions, it lacks structural and policy-level integration. Safety is strongly emphasized in policy but less so in research, and timeliness is addressed mainly through access and early detection without standardized metrics.

**Findings from Phase 2 (Interest Group Interviews)- Unpublished**

Al Hamidh, F., Noor, F., Rahman, MH., Alam, Z., Al Bedwawi, F., Shea, C., Nurelhuda, N. *Perspectives of Interest Groups on Health Quality Management Practices for Cardiovascular diseases, Diabetes, and Obesity in the United Arab Emirates: A Qualitative Exploration*. Unpublished manuscript.

Interest group interviews highlight a more practice-oriented and system-level perspective, revealing gaps between policy intentions and real-world implementation. Interest groups emphasized strong governance and leadership commitment, but identified significant challenges in policy translation, accountability, and coordination across sectors. Collaboration exists across governmental, academic, and service delivery domains; however, it remains fragmented and insufficiently integrated.

From a structural standpoint, stakeholders reported inequities in financing and insurance coverage, particularly affecting prevention and access to care. Workforce challenges emerged prominently, including capacity limitations, skill gaps, workload pressures, and insufficient training, alongside gaps in patient-centered communication. Health information systems were described as fragmented and insufficiently integrated, despite the growing role of digital technologies in supporting care delivery, monitoring, and patient engagement.

At the process level, participants highlighted significant fragmentation in care delivery, including weak coordination across settings, poor continuity of care, and gaps in patient follow-up systems. While there is an emerging shift toward patient-centered and adaptive care models, implementation remains inconsistent, with persistent challenges in patient engagement, health literacy, and communication. Societal partnerships are evolving, with increasing emphasis on multi-sectoral collaboration and community engagement, yet these efforts are hindered by limited coordination and accountability mechanisms.

Regarding outcomes, stakeholders identified multiple systemic barriers to effectiveness, including gaps in workforce capacity, policy implementation, and patient engagement. Efficiency is constrained by fragmented care pathways, resource limitations, and digital usability challenges, although digitalization and data-driven governance are seen as key enablers. Equity concerns were strongly emphasized, particularly in relation to insurance coverage, resource distribution, and population representation. Safety systems are generally well established, but gaps remain in safety culture, education, and workforce training. Timeliness of care is affected by capacity constraints and fragmented processes, although improvements are noted through digital and integrated care models.
