# Supplementary material for: Strengthening health quality management for cardiovascular disease, diabetes, and obesity in the United Arab Emirates: an integrated evidence synthesis
Source: Front Public Health. 2026 Oct 28;14:1918436. doi: 10.3389/fpubh.2026.1918436 (PMC13752134; doi:10.3389/fpubh.2026.1918436)
Supplement: Supplementary file 2 [file Table_1.docx]

Supplementary Material

# ENTREQ Checklist — Phase 3 Integrated Evidence Synthesis

| **S.N** | **ENTREQ item** | **How the item is addressed in the manuscript** | **Reported under section** |
| --- | --- | --- | --- |
| 1 | **Aim** | Synthesizing evidence generated from Phase 1 (scoping review) and Phase 2 (interest-group interviews) to identify systemic gaps, challenges, and opportunities for strengthening HQM for CVD, diabetes, and obesity care in the UAE. | 1- **Introduction** |
| 2 | **Synthesis methodology** | An integrative evidence synthesis using thematic synthesis and data triangulation was undertaken. The analysis was guided by an integrated HQM framework combining Donabedian, IOM quality dimensions, and WHO health-system building blocks expanded by Sacks. A mixed deductive–inductive approach was applied. | **2.2- Analytical approach for evidence synthesis** |
| 3 | **Approach to searching** | Literature search was undertaken as part of the preceding Phase 1 scoping review. Phase 3 used the findings generated from the completed scoping review rather than undertaking a new literature search. | **2.1- Study design and data extraction; Table 1** |
| 4 | **Inclusion criteria** | The eligibility scope of both evidence streams is summarized in Table 1. Phase 1 included evidence addressing HQM practices related to CVD, diabetes, or obesity in the UAE; Phase 2 included key informants with relevant expertise and knowledge of NCD program development and implementation. | **Table 1** |
| 5 | **Data sources** | Phase 1 used PubMed, Scopus, ProQuest and relevant national and international grey-literature sources. Phase 2 participants were identified through interest-group mapping and prioritization. The two evidence streams comprised 54 Phase 1 sources and 20 Phase 2 key informants. | **2.1- Study design and data extraction; Table 1** |
| 6 | **Electronic search strategy** | The electronic search strategy was undertaken and reported in the previously published Phase 1 scoping review by Alam et al.; no new electronic search was undertaken for Phase 3. | **2.1- Study design and data extraction; Reference 13 (Alam et al., 2026)** |
| 7 | **Study screening methods** | Study screening was conducted as part of the previously published Phase 1 scoping review and was not repeated during Phase 3. | **Reference 13 (Alam et al., 2026)** |
| 8 | **Study characteristics** | Characteristics of the two evidence streams are summarized in Table 1, including study/design, evidence type, period, sources/identification, eligibility scope, evidence/sample included, and geographical coverage. | **Table 1** |
| 9 | **Study selection results** | Study identification and selection were undertaken and reported in the published Phase 1 scoping review. Phase 3 used the resulting evidence base of 54 sources. | **2.1- Study design and data extraction; Reference 13 (Alam et al., 2026)** |
| 10 | **Rationale for appraisal** | No additional formal quality appraisal was undertaken as part of Phase 3; the synthesis used reported findings generated from the two preceding evidence streams. | **2.1- Study design and data extraction** |
| 11 | **Appraisal items** | No additional appraisal tool or criteria were applied to the two evidence streams during Phase 3. | **2.1- Study design and data extraction** |
| 12 | **Appraisal process** | No additional formal appraisal process was undertaken during Phase 3. | **2.1- Study design and data extraction** |
| 13 | **Appraisal results** | Evidence was not excluded or formally weighted on the basis of a new Phase 3 quality assessment. The two streams were interpreted according to their respective evidentiary roles. | **2.1- Study design and data extraction** |
| 14 | **Data extraction** | Reported findings from the Phase 1 scoping review and Phase 2 interest-group interviews were analyzed. Results from the two evidence streams were coded separately and organized within a structured evidence-integration matrix before integration. | **2.1- Study design and data extraction; Supplementary Document 2** |
| 15 | **Software** | Phase 2 interview findings were initially coded through NVivo™. Phase 3 synthesis was subsequently conducted manually using a mixed deductive–inductive approach. | **2.2.2- Coding and thematic synthesis** |
| 16 | **Number of reviewers** | Two researchers (FA and MHR) collaboratively conducted the coding and synthesis. Codes and themes were reviewed together, and disagreements were resolved through discussion and iterative refinement until consensus. | **2.2.2- Coding and thematic synthesis** |
| 17 | **Coding** | Findings from both evidence streams were coded using a mixed deductive–inductive approach. Deductive coding was guided by the integrated HQM framework, while inductive coding allowed findings outside predefined domains to be retained and incorporated into emergent and cross-cutting themes. | **2.2- Analytical approach for evidence synthesis** |
| 18 | **Study comparison** | Sub-themes were initially developed separately for each evidence stream and subsequently compared through triangulation to identify convergence, complementarity, and divergence. | **2.2.2- Coding and thematic synthesis**  **Braun and Clarke’s step # 3** |
| 19 | **Derivation of themes** | Theme development followed a mixed deductive–inductive process guided by Braun and Clarke's six steps. Integrated themes were reviewed across predefined HQM domains, with recurrent and emergent findings clustered into broader cross-cutting thematic areas. | **2.2.2- Coding and thematic synthesis**  **Braun and Clarke’s steps** |
| 20 | **Quotations** | Phase 3 synthesized **reported findings** from the two preceding evidence streams rather than re-analyzing raw primary interview data or primary-study data; therefore, primary participant/study quotations were not used as the principal synthesis output. | **N/A** |
| 21 | **Synthesis output** | The synthesis generated eight cross-cutting thematic areas and integrated themes, deriving gaps, challenges and recommendations. | **3-Results** |
